# TreeWatch.net: A Water and Carbon Monitoring and Modeling Network to Assess Instant Tree Hydraulics and Carbon Status

**DOI:** 10.3389/fpls.2016.00993

**Published:** 2016-07-05

**Authors:** Kathy Steppe, Jonas S. von der Crone, Dirk J. W. De Pauw

**Affiliations:** ^1^Laboratory of Plant Ecology, Department of Applied Ecology and Environmental Biology, Faculty of Bioscience Engineering, Ghent UniversityGhent, Belgium; ^2^Phyto-ITGhent, Belgium

**Keywords:** sap flow, stem diameter variation (dendrometer), process-based modeling, vegetation modeling, turgor, hydraulic failure, plant growth, drought

## Abstract

TreeWatch.net is an initiative that has been developed to watch trees grow and function in real-time. It is a water- and carbon-monitoring and modeling network, in which high-quality measurements of sap flow and stem diameter variation are collected on individual trees. Automated data processing using a cloud service enables instant visualization of water movement and radial stem growth. This can be used to demonstrate the sensitivity of trees to changing weather conditions, such as drought, heat waves, or heavy rain showers. But TreeWatch.net’s true innovation lies in its use of these high-precision harmonized data to also parameterize process-based tree models in real-time, which makes displaying the much-needed mechanisms underlying tree responses to climate change possible. Continuous simulation of turgor to describe growth processes and long-term time series of hydraulic resistance to assess drought-vulnerability in real-time are only a few of the opportunities our approach offers. TreeWatch.net has been developed with the view to be complementary to existing forest monitoring networks and with the aim to contribute to existing dynamic global vegetation models. It provides high-quality data and real-time simulations in order to advance research on the impact of climate change on the biological response of trees and forests. Besides its application in natural forests to answer climate-change related scientific and political questions, we also envision a broader societal application of TreeWatch.net by selecting trees in nature reserves, public areas, cities, university areas, schoolyards, and parks to teach youngsters and create public awareness on the effects of changing weather conditions on trees and forests in this era of climate change.

## Introduction

Climate change is impacting forests worldwide, threatening biodiversity, and ecosystem function and services (Anderson-Teixeira et al., 2015). Biological consequences of climate change are already apparent (Hughes, 2000), including increased tree mortality through drought, heat stress, insect infestation, and disease outbreaks (Anderegg et al., 2015; Teskey et al., 2015). Just as internationally coordinated forest monitoring initiatives boosted in the 1970s to respond to urgent scientific, political, and societal questions related to forest decline in relation to air pollution (Rautio and Ferretti, 2015), large monitoring networks around the globe (**Table 1**) are now investigating forest ecosystem responses to climate change. In particular, understanding the biological response of forests to climate change remains a great challenge, but is critical to biodiversity conservation, and management of ecosystem function and services (Anderson-Teixeira et al., 2015). Data sets from various monitoring initiatives and model forecasts are two essential components to no only understand forest ecosystem responses to climate change, but are also essential to support forest decision makers (Lindner et al., 2014). Adaptation strategies need a framework that includes monitoring and modeling activities as otherwise the forest manager will be grappling in the dark when making decisions (FAO, 2012).

**Table 1:** Important international large-scale forest monitoring networks.

Initiative	Founded	Extent	Goals and approach
**IUFRO^a^** International Union of Forest Research Organizations: the Global Network for Forest Science	1892	International 110 countries 700 member organizations 15000 scientists	IUFRO is a worldwide international organization devoted to forests and related sciences. IUFRO promotes excellence and knowledge sharing in forest-related research to enhance the understanding of ecological, economic and social aspects of forests and trees. IUFRO formulates forest-related policies, and supports ‘supersites’, which are global networks where state-of-the-art instruments are being used to obtain long-term baseline data.

**CTFS-ForestGEO^b^** Center for Tropical Forest Science-Forest Global Earth Observatory	1980	International with focus on tropical regions 24 countries 63 forest research plots 200 scientists	CTFS-ForestGEO is a unified, global network of forest research plots and scientists dedicated to the study of forest function and diversity with a strong focus on tropical regions. CTFS-ForestGEO aims at conducting long-term research on forests in order to increase understanding of forest ecosystems, monitor the impacts of global change, guide sustainable forest management and build capacity in forest science. Participating research sites are continuously monitored using a standardized tree census protocol, typically repeated every five years. Primarily diameter at breast height is measured, and changes in biomass or productivity, demographic rate and community composition are characterized.

**ICP Forests^c^** International Co-operative Program on assessment and monitoring of air pollution effects on forests	1985	International 42 countries 6000 level I plots 800 level II plots	ICP Forests is one of the largest biomonitoring networks launched in response to wide public and political concern about extensive forest damage. ICP Forests provides a periodic compilation of spatial and temporal variation of forest condition (level I), and aims at a better understanding of the cause-effect relationships between the condition of the forest and stress factors (level II) in Europe and beyond. Extensive level I monitoring provides an annual overview on forest condition based on defoliation, discolouration and visible damage on trees. Intensive level II monitoring includes more frequent surveys of crown condition (every year), foliar chemistry (every 2 years), soil chemistry (every 10 years), tree growth (every 5 years), ground vegetation (every 5 years), atmospheric deposition (continuous), and meteorology (continuous). Harmonized methods for sampling and analysis^d^ are adopted across all plots.

**FLUXNET^e^**	1996	International 64 countries 827 tower locations of which 526 are active	FLUXNET exists of regional networks, and coordinates regional and global analysis of observations from micrometeorological tower sites. FLUXNET provides infrastructure for a central database of site characteristic data, supplies information about the availability of flux data and compiles, archives and distributes carbon, water and energy flux measurements. Flux tower sites use eddy covariance methods to measure the exchange of CO_2_, water vapor and energy between terrestrial ecosystems and the atmosphere at 30-min frequency.

**ILTER Network^f^** International Long Term Ecological Research Network	1980 (US LTER) 1993 (ILTER) 2003 (LTER-Europe)	International (ILTER): 41 countries (formal LTER/LTSER) 5 countries (candidate, potential or at risk) US^g^: 26 LTER sites 2000 scientists Europe^h^: 25 countries 400 LTER sites 35 LTSER platforms	ILTER has been created to conduct research on ecological issues, which can last for decades, and spans huge geographical areas. ILTER brings together national or regional networks of scientists, engaged in long-term, site-based ecological (LTER) and socio-economic (LTSER) research to improve our knowledge on the structure and functions of ecosystems. One of the founding members of ILTER is the Chinese Ecosystem Research Network (CERN), which has been established in 1988, and is currently one of the largest national networks in the world. ILTER provides scientific expertise, research platforms, and long-term datasets to document and analyze environmental change. ILTER contributes to the knowledge base informing policy and to the development of management options in response to the grand challenges under global change.

**ICOS RI^i^** Integrated Carbon Observation System Research Infrastructure	2008	Pan-European 8 countries 95 measuring stations	ICOS RI provides harmonized and high precision scientific data on carbon cycle and greenhouse gas budget, and perturbations, which is openly available. ICOS RI was created to establish a sustained greenhouse gas observation system and to enable high quality climate change research. It provides long-term observations required to understand the present state, and to predict the future behavior, of the global carbon cycle and greenhouse gas emissions. ICOS RI installs standardized and integrated national atmospheric (CO_2_, CH_4_, CO, and radiocarbon-CO_2_ concentrations), ecosystem (fluxes of CO_2_, CH_4_, H_2_O, and heat) and marine (surface ocean – atmosphere carbon exchange, acidification, temperature…) stations.

*Each initiative is briefly defined, and main goals and approaches have been summarized. The year of foundation and current extent are also given. Networks are sorted from oldest to newest.*

^a^*http://www.iufro.org*

^b^*http://www.forestgeo.si.edu (Anderson-Teixeira et al., 2015)*

^c^*http://icp-forests.net*

^d^*http://icp-forests.net/page/icp-forests-manual*

^e^*http://fluxnet.ornl.gov*

^f^*http://www.ilternet.edu*

^g^*https://www.lternet.edu*

^h^*http://www.lter-europe.net*

^i^*https://www.icos-ri.eu*

While data from the larger networks (**Table 1**) add to the abundant evidence that forests globally are changing, it remains difficult to identify the mechanisms underlying such changes (Anderson-Teixeira et al., 2015). Focusing on responses of individual trees could single out the relative contributions of these underlying mechanisms. Indeed, trees have been named the ‘living laboratories’ for climate change responses (Farrell et al., 2015). A tree, like a human, can be viewed as a complex organism with an array of regulatory mechanisms to keep critical systems operating within appropriate bounds and mechanisms to repair damage that may occur when these bounds are exceeded (Anderegg et al., 2012).

In existing networks (**Table 1**), individual tree measurements are, despite their enormous potential, limited to incremental growth, which is conventionally measured every 3–5 years with a tape measure or with simple band dendrometers to track changes in stem circumference. These tree readings can help to start answering major questions about climate change and the potential uptake of CO_2_ emitted by human activity, but how carbon sequestration and the size of the carbon sink will alter with climate change remains highly uncertain (Popkin, 2015). Because radial stem growth is highly dynamic, species-specific, and dependent on environmental factors (see Köcher et al., 2012, 2013), it is a good indicator of tree vitality and of tree responses to environmental stress (Dobbertin, 2005). Modern systems connected to data loggers monitor changes in stem radius (or diameter) at high-frequency time resolution (minute scale), and use these as biological drought and growth indicator (e.g., TreeNet^1^, Zweifel, 2016). In addition, a close relationship with net ecosystem productivity, which integrates fluxes over the entire forest ecosystem, has been observed, and although causal explanation for this strong relationship is still fragmentary, it points to a compelling complementarity between both monitoring systems (Zweifel et al., 2010; Gea-Izquierdo et al., 2014).

Continuous time series of stem diameter variations capture diel tree water relations (reflected in reversible shrinkage and swelling) superimposed onto (long-term) irreversible radial stem growth (Steppe et al., 2006; Zweifel et al., 2006; De Swaef et al., 2015). Indeed, changes in diameter result from interactions of water and carbon inside the tree stem (Steppe et al., 2015a). These changes are a great source of tree physiological and ecological information (Zweifel, 2016), which can assist disentangling the mechanisms underlying forest ecosystem responses to climate change. In this paper, we therefore draw on the analogon of an intensive care unit in medical sciences to propose an approach to assess in real-time the tree’s trim by considering the tree as a complex organism that transports water from the soil to the atmosphere and sugars from the leaves (sources) to other plant organs (sinks), and whose survival hinges on maintenance of both transport systems. Monitoring equipment on the individual tree level is then used in combination with process-based modeling to translate raw sensor readings into physiological relevant measures, and to simulate key variables, which might be difficult to measure otherwise. We argue that an integrated approach that considers both tree monitoring and process-based modeling is needed to accurately predict forest dynamics in a changing climate.

Smart selection of a certain number of trees in a forest, and of different species in a stand, across different locations and ecosystems over longer time periods, will provide insight into growth, survival and adaptation strategies at the tree and ecosystem scales. The selection of trees and their up-scaling can be inspired by studies that have successfully scaled-up water use from tree to stand level (e.g., Granier et al., 1996; Köstner et al., 1998; Gebauer et al., 2012). Continuous measurements and modeling on the individual tree level are currently lacking in existing networks (**Table 1**) and are largely absent from process-based ecosystem models, while they can provide vital information on internal physiology of tree hydraulic and carbon status complementary to large-scale fluxes measured by eddy covariance and will better inform projections of forest ecosystem responses to climate change.

## The Treewatch.net Initiative

### Tree Monitoring

**FIGURE 1:**
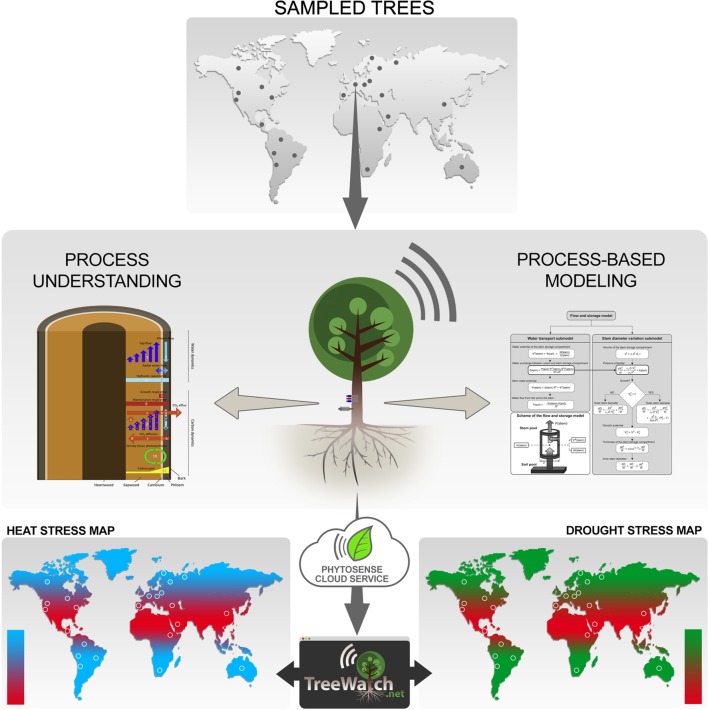
**Sketch of the TreeWatch.net approach.** Trees are typically sampled in natural forest ecosystems across the globe (dots represent fictitious sampling locations), but can also be city trees in urban settings. Sampled trees are equipped with a ‘heartbeat’ monitor, which currently consists of a sap flow sensor and a stem diameter variation sensor. The internet-connected plant sensors send their data to the PhytoSense cloud service, which handles data storage, data analysis, data processing, running of process-based model simulationsand calibrations, and sending out notifications. This all happens in real-time, which enables TreeWatch.net, a website built on top of the PhytoSense cloud service, to report instantly on each monitored tree’s health status. The unique approach of combining continuous tree measurements with process-based modeling lays the ground for the next-generation global maps displaying direct biological responses of the sampled trees; information which is currently lacking to bridge the gap with meteorology.

Climate change is expected to drive important changes in tree physiology with manifold but not yet fully understood impacts on forest ecosystem function and services. In this paper, we strongly support the recent calls to focus experimental, observational, and modeling efforts on the tree level to improve our understanding of climate change impacts on forests (Fatichi et al., 2014; Steppe et al., 2015a). We therefore view the tree as a complex organism that needs monitoring equipment to capture a ‘heartbeat’, which informs us on its actual trim. In contrast to humans, a tree does not have a real heartbeat, but continuous measurements with plant sensors on the tree stem display periodic signals that resemble a human’s electrocardiogram (Steppe et al., 2015a). These signals inform us on changes in plant hydraulics and carbon metabolism in xylem and phloem tissues (Steppe et al., 2015a; Zweifel, 2016). From the reviewed methods to quantify real-time water and carbon dynamics within a tree stem (Steppe et al., 2015a), a set of two sensors has currently been selected as basic monitoring equipment: a sap flow sensor and a stem diameter variation sensor (**Figure 1**). When combined with mechanistic modeling (Section “Process-Based Tree Modeling”), these measurements allow revealing the internal tree hydraulics and carbon status. As science evolves, other monitoring equipment, such as acoustic emission sensors (De Roo et al., 2016), may be added or may replace existing ones.

Sap flow is measured with a sap flow sensor, which uses heat to sense water movement in the stem xylem and is typically expressed as sap flow rate (in g h^-1^; Smith and Allen, 1996; Steppe et al., 2010; Vandegehuchte and Steppe, 2013). Accurate estimates of sap flow are essential in our tree monitoring approach to assess changes in tree hydraulics, internal water storage dynamics, and to quantify whole-tree water use, but also to estimate the tree’s carbon budget and stem respiration based on measurements of xylem CO_2_ transport and stem CO_2_ efflux (Teskey et al., 2008; Steppe et al., 2015a).

Point dendrometers or linear variable displacement transducers (LVDTs) measure variations in stem diameter (mm) at high temporal resolution (minute scale). The sensor signal simultaneously displays the integrated result of: (i) irreversible radial xylem and phloem growth; (ii) reversible shrinking and swelling of the living stem cells due to changes in internally stored water; (iii) contraction and expansion of dead conducting xylem elements due to the increase and relaxation of internal tensions; and (iv) thermal expansion and contraction of the stem (Daudet et al., 2005; De Swaef et al., 2015). Because of the tight coupling between tree hydraulics and radial stem growth and, hence, carbon metabolism, variations in stem diameter are the second vital component in our tree monitoring approach.

### Process-Based Tree Modeling

Current spatiotemporal knowledge of climate-forest dynamics is primarily based on simulations by dynamic global vegetation models (DGVMs). Although turgor, or the positive pressure potential in living cells, is the critical component in quantifying growth (Lockhart, 1965; Génard et al., 2001; Steppe et al., 2006), all existing DGVMs simulate long-term tree and forest stand growth using photosynthesized net carbon as source, which is then, according to allometric rules or simplified functional allocation schemes, partitioned among different carbon pools without considering tree hydraulics. Therefore, Fatichi et al. (2014) correctly call for moving from such a carbon source to a more sink-driven vegetation modeling in which water transport and turgor play a key role. As discussed previously (Steppe et al., 2006, 2015a), turgor is not only affecting cell wall expansion or irreversible radial growth (Lockhart, 1965), but also other growth processes, such as cell formation, deposition and assembly of new cell wall material depend on turgor and cell volume (Boyer, 1968; Ray, 1987; Proseus and Boyer, 2006). Because turgor in living tree cells is mainly built-up during night upon refilling of dehydrated tissues, growth processes mainly occur during the night, and are only optimal when tree water status is also optimal (Daudet et al., 2005; Saveyn et al., 2007; Steppe et al., 2015a). If we aspire a better spatiotemporal description of water fluxes together with more realistic scenarios for future climate and the carbon cycle (Friedlingstein et al., 2006; Thornton et al., 2007; Bonan et al., 2011), next generation DGVMs need to include a mechanistic understanding of water and carbon dynamics in xylem and phloem, and their interactions, at the tree level.

During the past few decades, implementation and application of process-based tree models has greatly advanced our knowledge on plant hydraulic functioning and growth (Steppe et al., 2015b). Started from a simple Ohm’s law analog model proposed by van den Honert (1948) for steady-state water transport, process-based tree models have greatly improved since, and large efforts have recently being put into the integration of xylem and phloem transport pathways, given the important coupling between hydraulic processes and the transport and allocation of carbohydrates (Höttlä et al., 2009; De Schepper and Steppe, 2010; Hubeau and Steppe, 2015). To further our knowledge of climate change impacts on the forest scale, process-based tree models are likely to become increasingly important.

In our approach, we advocate a combination of process-based tree modeling and continuous measurements at the tree level to better understand impacts of climate change on forests. Given that the current ‘heartbeat’ tree monitor consists of sap flow and variation in stem diameter, any process-based model that interlinks both processes is a direct candidate for our framework. The history and the current-state-of-the-art of possible candidate process-based models have recently been reviewed (De Swaef et al., 2015). The models we typically consider simulate tree sap flow dynamics, which can be directly linked to variations in stem diameter by using radial flow of water between xylem and phloem (**Figure 1**). The radial water flow causes changes in stem water content via a hydraulic capacitance, which results in changes in turgor, and drives irreversible radial stem growth according to Lockhart’s (1965) equation on top of elastic shrinkage and swelling. Steppe et al. (2006) originally developed such a so-called flow and storage model. Of particular interest for our approach is that such models feature essential hydraulic parameters (resistance and capacitance), and enable simulation of vital, but often difficult to measure variables (earlier described turgor, water potential), which all play an important role in hydraulic failure, tree mortality, and, therefore, long-term forest dynamics (Fatichi et al., 2014).

### Phytosense Cloud Service

Whereas continuous tree measurements, including sap flow and stem diameter variation, have been recognized as promising technology for monitoring tree hydraulics and carbon status (Anderegg et al., 2012; Köcher et al., 2013; De Swaef et al., 2015; Steppe et al., 2015a; Zweifel, 2016), and the use of process-based tree models is expected to get a boost given the recent recommendations on next-generation DGVMs (McDowell et al., 2013; Fatichi et al., 2014), no existing framework combines continuous tree readings with mechanistic modeling in real-time. This is exactly what our approach is aiming at: instant information on tree hydraulics and carbon status using continuous measurements and process-based model simulations.

To optimally combine the continuous tree measurements with the process-based simulation models, and to ensure real-time visualization of the tree’s hydraulic function and carbon status, the PhytoSense cloud service is used in our approach (**Figure 1**). This cloud service is the ‘brain’ behind the commercial plant monitoring system PhytoSense^2^ and is responsible for real-time data storage, data analysis, data processing, running model simulations and calibrations, and sending out notifications.

All processing on the cloud service is performed automatically so that little or no user interaction is required. A powerful system based on ‘transformations’ has been developed for real-time conversion of raw into processed data. A wide range of transformations is available: averaging, cumulating, summing, integrating, filtering, minimum/maximum, and the ability to apply any arbitrary equation to the data. More advanced transformations are also available to calculate sap flow rates in real-time, and to automatically remove disturbances from diameter variation signals. Once defined, transformations are automatically applied each time new data is received. Besides transformations, PhytoSense also allows to run dynamic simulation models in real-time. Although not required, models are typically first implemented in the plant modeling software PhytoSim^3^ and then converted into optimized code, which can run on the PhytoSense platform. These models can be any set of algebraic and (first order) differential equations [see for instance Steppe et al. (2006) or De Swaef et al. (2015)], and can be automatically recalibrated at certain time intervals using a moving window calibration procedure [see also Steppe et al. (2008)]. This lays the ground for novel stress detection approaches and ecophysiological warning systems, because daily estimates of the calibrated model parameters can now be displayed as time series in real-time from which important tree physiological behavior can be derived. Finally, notifications can be generated when measured or simulated data is below or above a threshold value for a specified amount of time, when a sensor is offline for a specified amount of time or when a model parameter exceeds the appropriate bounds.

PhytoSense provides a flexible API (Application Programming Interface) that allows any internet-connected device to connect to it. Any online data logger can use the API to send data to PhytoSense and custom-build applications or websites can use the API to visualize the available data. This makes the data from the TreeWatch.net trees readily available, which fits the ‘Internet of Things’ vision of this era.

### TreeWatch.net and the Way Forward

TreeWatch.net^4^ is a website built on top of the PhytoSense cloud service (Section “Phytosense Cloud Service”; **Figure 1**). TreeWatch.net originated at the Laboratory of Plant Ecology, Ghent University, Belgium, to show how the combination of tree monitoring and process-based modeling can significantly contribute to instant assessment of stress impacts on tree hydraulics and carbon status. The unparalleled enthusiasm and interest of the COST STReESS^5^ community in such a measuring/modeling framework gave the impetus to develop TreeWatch.net further as a global water and carbon monitoring and modeling network for advanced research on the dynamic interplay between trees and the regional climate.

Currently, TreeWatch.net monitors beech (*Fagus sylvatica* L.) and oak (*Quercus robur* L.) trees in the experimental forest Aelmoeseneie of Ghent University, Belgium. Sap flow is measured with custom-built Sapflow+ sensors (Vandegehuchte and Steppe, 2012) and stem diameter variations are recorded with a point dendrometer (model ZN11-T-WP, Natkon, Switzerland). We plan to gradually extend the network by adding trees across north-south trajectories in different populations in Europe, and in other continents, to profit from a wide climatic gradient going from low temperatures in the northern sites to warm and dry conditions in the southern sites, where tree responses are expected to be temperature- and drought limited, respectively. Trees will be sampled according to a stringent protocol taking into account various tree characteristics (e.g., tree status, tree height, stem diameter, and leaf area), and will be equipped with standardized plant sensors to avoid variability in the collected data due to different sensor types. Sensors connected to data loggers with wireless data transfer and remote control accessibility are used to send the data to the PhytoSense cloud service. The harmonized data offered by TreeWatch.net will be used in an innovative way to parameterize real-time process-based tree models (Section “Process-Based Tree Modeling”), and to run the models to understand tree response to climate change and growth differences across trajectories from underlying water and carbon relationships. Modeling will enable us to put the continuous measurements in a larger context by helping us understand the more general concepts underlying growth and tree hydraulic functioning.

Continuous real-time model simulations of the much-needed turgor when aspiring growth modeling, but also dynamics in model parameters, including hydraulic resistance and capacitance, are only a few of the opportunities that will be at hand to perform an integrated survey of tree responses to changes in the regional climate. These modeled features should be validated with ground-based data from fieldwork to increase confidence in the model, or to further improve it when discrepancies between modeled and measured data are observed. By visualizing hydraulic features, like hydraulic resistance, we will be the first to show changes in tree hydraulics and vulnerability to drought stress in real-time. The real-time aspect is a much-needed feature because now science relies on off-line, destructively collected vulnerability curves (Choat et al., 2012), which makes assessment indirect and therefore less reliable. In addition, TreeWatch.net aims at displaying pioneering maps with biological tree response to temperature and drought (**Figure 1**), which will be used to bridge the gap between tree functioning and meteorology (weather formation). Weather station data and soil moisture status at the sites, which are needed to interlink tree responses and regional climate, can be either additionally measured or accessed from existing networks (**Table 1**).

The results from TreeWatch.net are expected to spur discussion regarding long-standing assumptions for relationships between fluxes observed at the ecosystem level and the mechanisms responsible. Especially in DGVMs, the use of coarse scale observations and potentially incorrect mechanisms could mislead mitigation and adaptation plans of the future (Hanson and Gunderson, 2009). TreeWatch.net is, therefore, developed to be complementary to the data from these larger networks (**Table 1**) and may help to identify the much-needed mechanisms underlying changes in forests. But the use of TreeWatch.net is not limited to research in natural forest ecosystems only, as it can also be used in cities and urban plantings where trees are known to grow in a ‘future climate’ and can be used as ‘living laboratories’ to study plant responses to climate change (Farrell et al., 2015). At present, a maple (*Acer pseudoplatanus* L.) tree monitored at the Faculty of Bioscience Engineering^6^, Ghent University, serves this purpose.

TreeWatch.net is not only powerful in science, but is also able to serve an important educational role by teaching youngsters about the role of trees as regulators of the environment through so-called ‘talking forests’^7^. In May 2015, the experimental forest of Ghent University was opened as such a ‘talking forest’, for which a dedicated website^8^ has been designed using the flexible API of the PhytoSense cloud service. Assisted by experienced nature guides, students from both primary and secondary schools can now be invited to ‘listen’ to the trees and can find the real-time measurements on their phones or tablets to get insight into the interaction between climate and the forest.

## Conclusion

TreeWatch.net primarily aims at addressing forest and environmental issues that are of concern for our society, and takes the challenge to provide answers to urgent scientific and political climate-change related questions. But because of its intrinsic educational power, one of the long-term dissemination perspectives of TreeWatch.net is that trees are also selected in nature reserves, MAB^9^-sites, public areas, cities, university areas, schoolyards, and parks to create global awareness on the effects of (extreme) weather conditions on trees growing across all continents.

## Author Contributions

KS initiated TreeWatch.net and designed the outline of the initiative. She directed the network toward simultaneous application of continuous tree measurements and process-based modeling. She established the first network in Belgium and added a city tree to demonstrate its potential. She supervises the practical work, the analysis and data interpretation, and she coordinates the modeling activities within the TreeWatch.net framework. DD developed the PhytoSense cloud service and supervises data acquisition, data processing and visualization. He also assists in modeling activities. JvdC assists in daily data inspection, data analysis and data interpretation. He also designed the website. KS wrote the manuscript, JvdC reviewed the existing networks, and JvdC and DD contributed by critically revising the draft. All authors approve the final version to be published.

## Conflict of Interest Statement

The authors declare that the research was conducted in the absence of any commercial or financial relationships that could be construed as a potential conflict of interest.

## Footnotes

1 http://www.treenet.info

2 Developed by Phyto-IT (Mariakerke, Belgium): http://www.phytosense.net

3 Developed by Phyto-IT (Mariakerke, Belgium): http://www.phyto-it.com/PhytoSim.shtml

4 http://treewatch.net

5 COST Action FP1106 – Studying Tree Responses to extreme Events: a SynthesiS: http://www.streess-cost.eu

6 http://treewatch.net/faculty-of-bioscience-engineering/

7 http://www.flanderstoday.eu/innovation/talking-forest-teaches-youngsters-about-regulating-environment

8 http://www.aelmoeseneiebos.ugent.be/?p=pratendbos

9 Man and the Biosphere
